# Cytotoxic T-lymphocyte-associated protein 4 +49A/G polymorphism in Down syndrome children with Hashimoto’s thyroiditis

**DOI:** 10.17305/bb.2022.7869

**Published:** 2023-08-01

**Authors:** Muhammad Faizi, Nur Rochmah, Yuni Hisbiyah, Rayi Kurnia Perwitasari, Qurrota Ayuni Novia Putri, Sukmawati Basuki, Anang Endaryanto, Soetjipto Soetjipto

**Affiliations:** 1 Faculty of Medicine, Department of Child Health, Dr. Soetomo General Hospital, Universitas Airlangga, Surabaya, East Java, Indonesia; 2 Faculty of Medicine, Universitas Airlangga, Surabaya, East Java, Indonesia; 3 Institute of Tropical Disease, Universitas Airlangga, Surabaya, East Java, Indonesia; 4 Department of Medical Biochemistry, Faculty of Medicine, Universitas Airlangga, Surabaya, East Java, Indonesia

**Keywords:** Down syndrome (DS), Hashimoto’s thyroiditis (HT), cytotoxic T-lymphocyte-associated protein 4 +49A/G (*CTLA-4* +49A/G)

## Abstract

Thyroid dysfunction is the most common endocrine disorder in Down syndrome (DS) children. Cytotoxic T-lymphocyte-associated antigen 4 (*CTLA-4*) is one of the immune regulatory genes that correlates with Hashimoto’s thyroiditis (HT). However, studies on *CTLA-4* +49A/G in DS children with HT are still limited. We aimed to evaluate *CTLA-4* +49A/G gene polymorphism in DS children with HT. This case-control study, conducted from February 2020 to February 2022 at Dr. Soetomo General Hospital, Surabaya, enrolled 40 DS children with HT and 50 healthy children. The DNA sequencing was performed to identify the polymorphism (Sanger sequencing). Thyroid peroxidase antibodies (TPOAb), thyroglobulin antibodies (TgAb), thyroid-stimulating hormone (TSH), and free thyroxine (FT4) levels were analyzed by enzyme-linked immunosorbent assay (ELISA). The mean age of DS children with HT was 1.78 years. Males predominated in the study population. Subjects with GG genotype were diagnosed earliest with hypothyroidism (8 months) compared with other studies. The most common thyroid dysfunction was central hypothyroidism, with TgAb positivity present in all patients. The AA genotype (odds ratio [OR] 0.265, 95% confidence interval [CI] 0.094–0.746; *P* ═ 0.012) and A allele (OR 0.472, 95% CI 0.309–0.721; *P* ═ 0.0002) were significantly more frequent in the control group. The AG genotype (OR 2.65, 95% CI 0.094–0.746; *P* ═ 0.003) and G allele (OR 2.116, 95% CI 1.386–3.23; *P* ═ 0.003) were more frequent in the DS with HT group. The age of the subjects in this study was younger than in previous studies. The AG genotype and the G allele were more prevalent in the DS with HT group and may be a risk factor in HT development in DS children. Furthermore, the AA genotype may act as a protective factor against HT in DS children.

## Introduction

Down syndrome (DS) is the most common chromosomal abnormality, occurring in 1 in over 800 births. It is mainly caused by the nondisjunction of chromosome 21, whereas the other cases are due to translocation or mosaicism [1–3]. According to the World Health Organization (WHO) report in 2018, approximately 3000−5000 children are born with chromosomal abnormalities each year [4]. There is an increased prevalence of DS in children from Indonesia, from 0.12% in 2010 to 0.21% in 2018, based on data obtained from the Ministry of Health in 2018 [5]. 

DS is associated with many abnormalities, such as pulmonary, gastrointestinal, cardiovascular, and endocrine problems, as well as developmental delay. Thyroid dysfunction is the most frequent endocrine disorder (4%–8%) in DS children [6]. Congenital hypothyroidism, subclinical hypothyroidism, hyperthyroidism, and acquired hypothyroidism (autoimmune and non-autoimmune) commonly occur in these children [7].

The prevalence of autoimmune thyroiditis increases in childhood due to genetic and environmental causes. Genetic factors are divided into two groups according to their functions: thyroid-specific genes and immune regulatory genes [8]. Cytotoxic T-lymphocyte-associated antigen 4 (*CTLA-4*) is one of the immune regulatory genes that are correlated with HT. Human *CTLA-4* is among the most researched loci (2q33) in terms of autoimmunity and its role in the development of Graves’ disease (GD) and/or Hashimoto’s thyroiditis (HT). CTLA-4 is an immunoregulatory molecule found on the surface of activated T-cells that inhibits T-cell activation by interacting with the B7 molecule [9]. *CTLA-4* single nucleotide polymorphism is correlated to the T-effector activity [10]. In particular, *CTLA-4* adenine–guanine polymorphism exon 1 position 49 (a peptide with a Thr/Ala exchange) single nucleotide polymorphism leads to the development of a faulty receptor, which reduces the inhibitory effect of CTLA-4 on T-cell activation and the important candidate for autoimmune thyroid diseases [11, 12, 52]. Antiperoxidase antibodies (TPOAb) and/or antithyroglobulin antibodies (TgAb) are the thyroid-specific antibodies observed in HT. The association between cytotoxic T-lymphocyte-associated protein 4 +49A/G (*CTLA-4* +49A/G) and HT has frequently been studied, whereas research on *CTLA-4* +49A/G in DS children with HT is still limited. This study aimed to evaluate the *CTLA-4* +49A/G in DS children with HT.

## Materials and methods 

### Study design

Forty DS children with HT who visited the Pediatric Endocrine Clinic at Dr. Soetomo General Hospital Surabaya from February 2020 to February 2022 and met the inclusion and exclusion criteria were enrolled in this case-control study. As a control group, 50 healthy children without a history of DS and/or HT were included. The consecutive sampling was used for selecting the study participants. The inclusion criteria were as follows: (a) DS children with HT (proven by the positivity of TPOAb and/or TgAb) aged 1 month to 18 years treated as outpatients at Dr. Soetomo General Hospital Surabaya; (b) DS confirmed by a karyotyping test; and (c) the patients’ parents signed the informed consent to study participation. DS children with HT who required Pediatric Intensive Care Unit care were excluded.

### DNA extraction and genotyping

The *CTLA-4* +49A/G (rs231775) was identified by DNA sequencing using the primer forward for *CTLA-4* +49A/G (rs231775) 5’-GCTCAGCTGAACCTGGCT-3’, and primer reverse 5’-AAATCACTGCCCTTGACTGC-3’. DNA was extracted from peripheral blood mononuclear cells by using the QIAmp mini Kit as instruction (Qiagen©, Germany). In total, the reaction volume of 10 µL (a primer set of *CTLA-4* +49A/G, Go Tag DNA polymerase [Promega, USA], 25 mM MgCl_2_, H_2_O, and 2 µL template DNA) was amplified with the initial denaturation at 92 ^∘^C for 3 min, then followed by 92 ^∘^C for 45 s, 58 ^∘^C for 1 min, 70 ^∘^C for 1 min (35 cycles) with the final extension at 70 ^∘^C for 3 min in the PCR machine (Labycler Sensequest Biomedizinische, Germany). The PCR product was evaluated using agarose gel 2% that runs in the electrophoretic field and resulted in a single band at 165 bp. The PCR product was purified with ExoSAP-IT (USB, USA). PCR sequencing was performed on 2 µL purified amplicons with a dye terminator (BigDye™ Terminator v3.1, Applied Biosystems, USA) and forward or reverse primer of *CTLA-4* +49A/G (rs231775). The PCR sequencing product was precipitated by ethanol solution, then dissolved in H_2_O and placed into a well plate. The filled well plate was put into the sequencer machine (ABI/Hitachi 3500XL, USA). This DNA sequencing was applied at the Institute of Tropical Diseases, Universitas Airlangga, Surabaya, Indonesia. The *CTLA-4* +49A/G (rs231775) polymorphism was examined by using FinchTV (Geospiza Inc, USA) and NCBI softwares.

### Thyroid function and antibody markers

Thyroid functions (free thyroxine [FT4], thyroid-stimulating hormone [TSH], TPOAb, and TgAb) were assessed using blood tests. The thyroid antibodies of HT were examined at the Institute of the Tropical Disease University of Airlangga, Surabaya. HT was postulated by the positivity of TPOAb and/or TgAb. The TPOAb values were classified as follows using a Demeditec kit (Demeditec Diagnostics GmbH, catalog number: DE7580): negative (<50 IU/mL); borderline (50–75 IU/mL), which was considered positive in this study; or positive (>75 IU/mL). The TgAb values were classified as follows using a Demeditec kit (Demeditec Diagnostics GmbH, catalog number: DE7590): negative (<100 IU/mL), borderline (100–150 IU/mL), which was considered positive in this study; or positive (≥150 IU/mL).

The thyroid function test results were analyzed at the Diagnostic Center of Soetomo General Hospital, Surabaya. The catalogue number for FT4 ELISA kit (Biochem Canada) was ‘CAN-FT4-4340’ and TSH Triiodothyronine was ‘CAN-TSH-4080’. The reference range values for pediatric care by Sperling et al. (2020) [13] were used to determine the normal range of TSH and FT4. Central hypothyroidism was defined by the low TSH and FT4 levels, primary hypothyroidism by the high TSH and low FT4 levels, subclinical hypothyroidism by the elevated serum TSH levels with normal FT4 levels, and euthyroidism by the normal FT4 and TSH levels [7]. 

### Ethical statement

This study was approved by Dr. Soetomo General Hospital Ethics Committee (Ref. No. 1960/KEKP/IV/26020). The authors’ institutional review board or comparable organization approved the present research for human subjects, which followed all relevant national rules, institutional procedures, and the precepts of the Helsinki Declaration.

### Statistical analysis

The SPSS 17 software was used to analyze all data. A descriptive analysis was performed on the distribution of the characteristics of participants and *CTLA-4* +49A/G (rs231775) polymorphism. The comparison of genetic polymorphism and allele distribution between DS patients with HT and the control group, thyroid dysfunction, TPO-Ab, and genetic polymorphism was analyzed with a chi-square test. Age at diagnosis of hypothyroidism and duration of therapy with levothyroxine compared with *CTLA-4* +49A/G (rs231775) polymorphism were analyzed with nonparametric Kruskal–Wallis H test. The regression test was analyzed with the logistic regression test. The significant result was defined as *P* < 0.05. The data are shown as mean and percentage (%). *P* value of Hardy–Weinberg Equilibrium (HWE) was also calculated.

## Results

### Characteristics of the study participants

The characteristics of the study participants are presented in Table 1. Forty DS children with HT and 50 healthy children voluntarily participated in this study. DS group with HT consisted predominantly of male participants, whereas the equal proportion of male and female participants was in the control group. Forty-five percent (18/40) of the DS children with HT had another congenital anomaly, such as congenital heart disease. FT4 and TSH levels in DS children with HT were 0.8 ± 0.5 ng/mL and 1.2 ± 6.6 µU/mL, respectively. All DS children with HT showed TgAb positivity.

**Table 1:** Characteristics of the study participants

**Characteristics**	**DS with HT**	**Control**
	**(*n* ═ 40)**	**(*n* ═ 50)**
Age (years), mean ± SD	1.78 ± 1.5	3.2 ± 2.27
*Sex, n (%)*		
Male	26 (65)	26 (52)
Female	14 (35)	24 (48)
*Thyroid function test, n (%)*		
Central hypothyroidism	31 (77.5)	–
Primary hypothyroidism	7 (14.6)	–
Subclinical hypothyroidism	2 (4.2)	–
*Value, n (%)*		
Positive for TgAb	40 (100)	–
Positive for both TPOAb and TgAb	26 (65)	–
Cumulative positive for autoimmune marker	40 (100)	–
*Value, mean ± SD*		
FT4 (ng/mL)	0.8 ± 0.5	–
TSH (µU/mL)	1.2 ± 6.6	–

Data presented as mean ± standard deviation (SD) or *n* (%). DS: Down syndrome; HT: Hashimoto’s thyroiditis; TPOAb: Thyroid peroxidase antibodies; TgAb: Thyroglobulin antibodies; FT4: Free thyroxine; TSH: Thyroid-stimulating hormone.

### *CTLA-4* + 49A/G (rs231775) gene polymorphisms in DS children with HT

Three kinds of genotypes were noted in the study participants. The details of *CTLA-4* +49A/G (rs231775) gene polymorphism in DS patients with HT and controls are described in Table 3. The AG genotype was the most common in DS children with HT. DS participants with the AG genotype had a 1.688 times higher risk of developing HT than those with other genotypes, although the results did not show statistical significance (odds ratio [OR] 1.688, 95% confidence interval [CI] 0.730–3.905; *P* ═ 0.221). The AA genotype was the most common in the control group. The AA genotype had a protective effect on DS children with HT 0.265 times compared and showed statistical significance (OR 0.265, 95% CI 0.094–0.746; *P* ═ 0.012). The G allele had increased risk for DS with HT (OR 2.116, 95% CI 1.386–3.23; *P* ═ 0.003). Furthermore, the A allele had protective effect (OR 0.472, 95% CI 0.309–0.721; *P* ═ 0.0002) (Table 2). Distribution consistent to Hardy–Weinberg’s law at level of significance 0.05.

**Table 2:** *CTLA-4* +49A/G gene polymorphism in DS children with HT at Dr. Soetomo General Hospital

**Variables**	**DS with HT (*n* ═ 40)**	**Control (*n* ═ 50)**	***P* value**	**OR (95% CI)**
Genotypes, *n* (%)			0.031	
AA	6 (15)	20 (40)	0.012*	0.265 (0.094–0.746)
AG	22 (55)	21 (42)	0.221	1.688 (0.730–3.905)
GG	12 (30)	9 (18)	0.185	1.952 (0.726–5.248)
Allele, *n* (%)				
A	68 (42.5)	122 (61)	0.0002*	0.472 (0.309–0.721)
G	92 (57.5)	78 (39)	0.0003*	2.116 (1.386–3.23)

Data presented as *n* (%). Logistic regression test, *P < 0.05 considered significant. DS: Down syndrome; HT: Hashimoto’s thyroiditis.; OR: Odds ratio; CI: Confidence interval; CTLA-4: Cytotoxic T-lymphocyte-associated protein 4.

**Table 3:** Correlation between thyroid dysfunction diagnoses and *CTLA-4* +49 A/G in DS children with HT

**Genotype**	**Central hypothyroidism**	**Primary hypothyroidism**	**Subclinical hypothyroidism**	***P* value**
AA, *n* (%)	4 (66.7)	2 (33.3)	0 (0)	0.667^a^
AG, *n* (%)	17 (77.3)	4 (18.2)	1 (4.6)	
GG, *n* (%)	10 (83.2)	1 (8.4)	1 (8.4)	

Data presented as *n* (%). ^a^Chi-square, *P* < 0.05 considered significant. DS: Down syndrome; HT: Hashimoto’s thyroiditis; CTLA-4: Cytotoxic T-lymphocyte-associated protein 4.

### The correlation between thyroid function diagnoses and *CTLA-4* +49A/G (rs231775) gene polymorphism in DS children with HT

This study showed that AG was the most frequent genotype found. All subjects having an AG genotype were commonly diagnosed with central hypothyroidism (77.3%), followed by primary (18.2%) and subclinical hypothyroidism (4.6%) even though not significantly correlated (Table 3).

### The correlation between TPOAb and *CTLA-4* +49A/G (rs231775) gene polymorphism in DS children with HT

The AG genotype was the most common genotype, and subjects with the AG genotype frequently had positive TPOAb. Also, the positivity of TPOAb showed dominance in other genotypes, even though TPOAb and *CTLA-4* +49A/G were not significantly correlated (*P* ═ 0.45). Otherwise, the correlation between TGAb and *CTLA-4* +49A/G gene polymorphism could not be done due to the constant result of TGAb positivity (Table 4).

**Table 4:** The correlation between TPOAb and *CTLA-4* + 49 A/G in DS children with HT

**Genotype**	**TPOAb**		***P* value**
	**Positive**	**Negative**	
AA, *n* (%)	4 (66)	2 (34)	0.45^a^
AG, *n* (%)	16 (73)	6 (27)	
GG, *n* (%)	6 (50)	6 (50)	

Data presented as *n* (%). ^a^Chi-square test, *P* < 0.05 considered significant. DS: Down syndrome; HT: Hashimoto’s thyroiditis; TPOAb: Thyroid peroxidase antibodies; CTLA-4: Cytotoxic T-lymphocyte-associated protein 4.

### Age at diagnosis of hypothyroidism and duration of therapy with levothyroxine compared with *CTLA-4* +49A/G polymorphism

There was no significant effect of age at diagnosis of hypothyroidism (*P* ═ 0.46), and duration of therapy with levothyroxine (*P* ═ 0.25) compared with *CTLA-4* +49A/G polymorphism (Table 5). Children with the GG genotype were diagnosed with hypothyroidism earlier than those with other genotypes.

**Table 5:** Age at diagnosis of hypothyroidism and duration of therapy with levothyroxine compared with *CTLA-4* +49A/G polymorphism

**Genotype**	**Age at diagnosis of hypothyroidism (months)**	***P* value**	**Duration of therapy with levothyroxine (months)**	***P* value**
AA, mean ± SD	18 ± 19.8	0.46*	6.7 ± 14.4	0.25*
AG, mean ± SD	11.4 ± 11.4		7.1 ± 14.8	
GG, mean ± SD	7.8 ± 7.7		17.3 ± 18.5	

Data presented as months, mean ± standard deviation (SD). *Kruskall–Wallis H test, *P* < 0.05 considered significant. CTLA-4: Cytotoxic T-lymphocyte-associated protein 4.

## Discussion

To the best of our knowledge, this is the first study that explored the *CTLA-4* +49A/G polymorphism in DS children with HT, as the studies on the association between CTLA-4 and HT are limited. We showed that the *CTLA-4* +49A/G gene polymorphism in DS children with HT was dominated by the AG genotype (55%), although not significantly differed from the control group (*P* ═ 0.221). Similar studies involving adult patients with HT but without DS in Poland and Slovakia (Caucasians), Turkey (Mixed), Japan and India (Asians) revealed that the most common genotype found in these patients was AG [15–22]. However, studies conducted in Thailand and Lebanon revealed that the most common genotype was AA [23, 24]. Meanwhile, Bicek et al. reported that GG is the most common genotype found in GD patients from Slovenia [25].

The AG genotype and the G allele had 1.688- and 2.116-fold higher risks, respectively, of developing DS with HT compared with other genotypes, similar to the results of some European and Asian studies [20, 22, 26, 27]. *CTLA-4* +49A/G increased the risk of HT among non-DS adults, particularly those with the AG genotype and G allele. The G49 allele was associated with HT, as confirmed by the higher thyroid autoantibody concentrations, including TgAb and TPOAb [28, 29]. According to previous studies, the G allele is associated with reduced control of T-cell proliferation, thereby contributing to the pathophysiology of autoimmune hypothyroidism, GD, and other autoimmune diseases [11].

Regarding chromosome 2q33, a susceptibility locus for thyroid autoantibody production was discovered around the marker D2S155, which is linked to the *CTLA4* and *CD28* genes [28]. Further research revealed that a significant gene for thyroid autoantibody production on chromosome 2q33 is most likely the *CTLA4* gene rather than the *CD28* gene [28]. 

Yung et al. reported that the G allele predominates in the Asian population compared to other ethnicities, whereas the A allele is commonly found in whites; moreover, the individuals with the AG genotype were reported to be more susceptible to developing HT [27, 30]. However, the patients in most case-control studies were ethnically matched, and both whites and Asians were found to have a link to the disease. As a result, ethnic and regional heterogeneities may only play a minor role in explaining discrepancies among the association studies [26]. Conflicting studies found no linkage between the *CTLA-4* gene and HT in Japan, Tunisia, and another Chinese pedigree [26, 31–33]. 

The AA genotype was significantly more prevalent in the control group in our study. The AA genotype as well as the A allele were significant protective factors against HT development. These results are consistent with those of European and Asian population-based studies [20, 26, 27]. 

The rate of GG genotypes in this study was not different between the DS patients with HT and controls. This result is contrary to Pastuszak et al.’s study involving GD patients from Poland and Patel et al.’s study involving non-DS patients from India; they reported that the GG genotype increased the risk for HT development in these patient groups [20, 22].

Congenital alteration in thyroid gland regulation among DS children is directly related to the trisomy of chromosome 21. The present study showed that central hypothyroidism was the most common diagnosis among DS children. This finding is different from those of other studies. Subclinical hypothyroidism was the most common thyroid dysfunction in DS children with HT, which is commonly asymptomatic and may not require treatment. However, subclinical hypothyroidism can progress to primary hypothyroidism associated with autoimmune factors [34–47]. TgAb positivity was more prevalent compared to TPOAb positivity, as observed in our study. A similar study by Gentile et al. demonstrated that TgAb was also more prevalent in HT patients [48]. Contrarily, Bjoro et al.’s study showed that TPOAb positivity was more frequently observed among HT patients, and it usually appears before thyroid dysfunction occurs, as compared to TgAb [48]. Thyroid autoimmunity affects DS children from an early age, but another study reported that thyroid autoantibodies start to appear at 5–8 years of age [34], indicating that autoimmune regression increases with age. Genetics has become a factor in autoimmune disorders, although the underlying reasons remain unknown [8].

The mean age of participants in this study was 1.78 years. Patients with GG genotype were diagnosed with hypothyroidism earliest compared with those with other genotypes and were younger compared with other studies, although there was no significant correlation. Studies by Aversa et al. stated that DS children develop thyroid dysfunction at 6.5 years, i.e., at a younger age than non-DS children, who develop thyroid dysfunction at 11 years old [49]. Similar to Sanyal and Bhattacharjee, the median age of DS children with hypothyroidism is younger than that non-DS children [50].

In our study population, the proportion of boys was greater than that of girls, which was similar to the findings of Musdalipa et al. and Kawanto et al. [46, 50]. However, Guaraldi et al. pointed out that there is no difference in the rate of HT between sexes [51]. 

In the present study, another congenital anomaly besides thyroid dysfunction, i.e., congenital heart disease, was present in 18 (45%) children with DS. A previous study showed that cardiovascular diseases are observed in 58% of DS children [51].

The major limitation of our study is that it was performed in only one health center and with limited sample size. We suggest performing the multicenter study on this topic.

## Conclusion

Central hypothyroidism is the most common thyroid dysfunction observed among DS children with HT. AG genotype and G allele of *CTLA-4* +49A/G gene polymorphism were frequently found in the patients’ group; these may be risk factors for the susceptibility to HT among DS children. Moreover, the AA genotype may play as a protective factor against HT in DS children.
